# Phenotypic effects of overexpression of the MMAC1 gene in prostate epithelial cells

**DOI:** 10.1054/bjoc.2000.1400

**Published:** 2000-10

**Authors:** R M Sharrard, N J Maitland

**Affiliations:** YCR Cancer Research Unit, Department of Biology, University of York, York, UK

**Keywords:** prostate cancer, MMAC1/PTEN/TEP1, cell survival, cell morphology

## Abstract

The prostate cancer cell lines PC3 and LNCaP have been shown to lack expression of the tumour suppressor gene MMAC1/PTEN, in contrast to the immortalized non-tumorigenic epithelial lines PNT1a and PNT2. We have measured the effects of reintroduction of wild type (wt) and mutant MMAC1 genes on to these genetic backgrounds, using gene constructs expressing either wt MMAC1 or various mutants deficient in the dual specificity phosphatase domain of the protein. Over-expression of wild type PTEN protein induced cell shrinkage and rounding, but did not result in increased levels of classical apoptosis. Permanently transfected lines containing the MMAC1 gene could only be obtained from the PNT cells, as PTEN expression resulted in rapid loss of both tumour lines. In contrast, mutation of the phosphatase domain resulted in partial attenuation of the phenotypic effects of MMAC1 after transient transfection, and also allowed the derivation of permanent tumour cell lines containing the mutated MMAC1 gene. The results suggest that re-expression of wt PTEN is incompatible with survival of human prostate cancer cells in vitro, and that the full biological activity of this common tumour suppressor requires functions additional to the established protein and lipid phosphatase activities in epithelial systems. © 2000 Cancer Research Campaign


*British Journal of Cancer* (2000) **83**(8), 1102–1109
© 2000 Cancer Research Campaign
doi: 10.1054/ bjoc.2000.1400, available online at http://www.idealibrary.com on IDEAL®

### Phenotypic effects of overexpression of the MMAC1 gene in prostate epithelial cells

**RM Sharrard and NJ Maitland**

YCR Cancer Research Unit, Department of Biology, University of York, York, UK

**Summary** The prostate cancer cell lines PC3 and LNCaP have been shown to lack expression of the tumour suppressor gene MMAC1/PTEN, in contrast to the immortalized non-tumorigenic epithelial lines PNT1a and PNT2. We have measured the effects of reintroduction of wild type (wt) and mutant MMAC1 genes on to these genetic backgrounds, using gene constructs expressing either wt MMAC1 or various mutants deficient in the dual specificity phosphatase domain of the protein. Over-expression of wild type PTEN protein induced cell shrinkage and rounding, but did not result in increased levels of classical apoptosis. Permanently transfected lines containing the MMAC1 gene could only be obtained from the PNT cells, as PTEN expression resulted in rapid loss of both tumour lines. In contrast, mutation of the phosphatase domain resulted in partial attenuation of the phenotypic effects of MMAC1 after transient transfection, and also allowed the derivation of permanent tumour cell lines containing the mutated MMAC1 gene. The results suggest that re-expression of wt PTEN is incompatible with survival of human prostate cancer cells in vitro, and that the full biological activity of this common tumour suppressor requires functions additional to the established protein and lipid phosphatase activities in epithelial systems. © 2000 Cancer Research Campaign

**Keywords:** prostate cancer; MMAC1/PTEN/TEP1; cell survival; cell morphology

Identification of preferential loci at which loss of heterozygosity (LOH) can be detected in prostate cancer is complicated by both inter- and intra-tumour heterogeneity (Bergerheim et al, 1991; Trybus et al, 1996; Macintosh et al, 1998). However, a consensus is emerging of the most frequent and significant loci (reviewed by Latil and Lidereau, 1998). One of the most frequently mutated loci is at chromosome 10q23 (Gray et al, 1995; Ittmann, 1996), and recent studies have implicated the gene MMAC1 (also known as TEP1 or PTEN) (Li et al, 1997; Steck et al, 1997) as a tumour suppressor gene at this locus. Direct determination of both LOH and mutation of MMAC1 have yielded results consistent with tumour suppressor function in prostate cancers (Cairns et al, 1997; Gray et al, 1998; Pesche et al, 1998; Suzuki et al, 1998). The locus, and by inference the MMAC gene, has also been implicated in other tumours such as glioblastoma, malignant melanoma, and carcinomas of the breast and endometrium (Li et al, 1997; Guldberg et al, 1997; Rhei et al, 1997; Tashiro et al, 1997; Boström et al, 1998).

Extensive in vitro studies in which the MMAC gene has been transferred into the MMAC-null glioblastoma cell line U87MG (Davies et al, 1998) have indicated that gene replacement can lead not to a restoration of normal cellular behaviour, but rather to cell death, linked to a loss of cell adhesion and motility (Tamura et al, 1998). With prostate cancers, the difficulty in introducing genes into prostatic epithelial cells has probably restricted this type of study to experiments on the LNCaP cell line, where apoptotic cell death was the observed result (Davies et al, 1999). Were such an effect to be biased towards tumour cells, then specific re-expression of the MMAC gene in prostate cancers could be a novel and effective tumour cytoreductive therapy.

The product of the MMAC locus, known most commonly as PTEN, has been shown to be a bifunctional protein and lipid phosphatase (Myers et al, 1997; Maehama and Dixon, 1998). The protein also has homologies to tensin and an actin binding domain, suggesting an association with cytoplasmic cytoskeletal elements such as focal adhesion plaques (Li et al, 1997; Tamura et al, 1998). The C-terminal half of PTEN, which has a regulatory influence over the phosphatase activity of the N-terminal domain (Georgescu et al, 1999), contains a C2 domain as well as presenting a good consensus PDZ binding domain at the extreme C terminus, both consistent with the membrane-binding capacity of this domain (Lee et al, 1999). A number of mutant variants which lack the primary phosphatase activity have been described (Furnari et al, 1997, 1998; Maehama and Dixon, 1998), and this activity has been directly related (again mainly in the glioblastoma system) to the observed biological activity (Cheney et al, 1998; Furnari et al, 1998; Myers et al, 1998; Maier et al, 1999). We have set out to study the relationship between the presence of PTEN protein and the behaviour of a series of prostatic epithelial cells including the LNCaP and PC3 tumour cell lines, which do not express PTEN protein, and the PNT cell lines, derived from normal prostatic epithelium, where we can demonstrate expression of normal PTEN. We show that, while cells expressing endogenous wt PTEN can tolerate small amounts of exogenous PTEN, neither of the tumour cell lines could survive introduction of the MMAC gene. In transient transfection assays using native or FLAG-epitope tagged PTEN protein, similar effects to those in glioblastoma cells on both adhesion and cell morphology were observed. However, phosphatase-inactivating mutations G129R

*Received 18 January 2000*
*Revised 13 June 2000*
*Accepted 17 June 2000*

*Correspondence to:* RM Sharrard and G129E only partially abrogated the biological effects, implying that domains other than the phosphatase directly modulate biological effects in prostatic epithelium.

### MATERIALS AND METHODS

### Cell lines

PNT1a and PNT2 (Cussenot et al, 1991; Berthon et al., 1995) are non-tumorigenic epithelial cells lines derived by SV40 immortalization of normal prostate epithelial outgrowths. LNCaP and PC3 are established prostatic carcinoma cell lines with androgen dependent and androgen independent phenotypes respectively, obtained from American Type Culture Collection. PNT1a, PNT2 and LNCaP cells were maintained in RPMI1640 containing 2 mM glutamine, 10 mM HEPES and 10% fetal calf serum (FCS); PC3 cells were maintained in Ham's F12 medium containing 2 mM glutamine and 7% FCS.

### Polymerase chain reaction (PCR)

1 μg of DNA was amplified in 50 μl reaction mixture containing 200 μM dNTPs, 1.5 mM $MgCl_2$, 0.7 μM of each primer, 1 × Expand buffer and 1 unit of Expand High Fidelity DNA polymerase (Boehringer). The primers used were: for exon 1, 5′-CAGCCGTTCGGAGGATTA-3′ and 5′-ATATGACCTAGCAACCTGACCA-3′; for exon 9, 5′-AAGATGAGTCATATTTGTGGGT-3′ and 5′-GACACAATGTCCTATTGCCAT3′. Amplification was carried out in a Perkin-Elmer 2400 thermal cycler according to the protocol: 2 min at 94°C; 30 cycles of 15 sec 94°C, 20 sec at 55°C and 75 sec + 2 sec/cycle at 72°C; 5 min at 72°C. PCR products were analysed by agarose gel electrophoresis, purified from the gel and directly sequenced.

### Reverse transcriptase-PCR (RT-PCR)

5 μg of total RNA from each cell line was primed with 0.25 μg oligo (dT) 12–15 (Life Technologies) and reverse-transcribed to cDNA in a 20 μl reaction containing 50 mM tris HCl pH 8.3, 75 mM KCl, 3 mM $MgCl_2$, 10 mM dithiothreitol, 1 mM dNTPs and 100 units Superscript II reverse transcriptase (Life Technologies), incubated at 45°C for 120 min. Equal volumes of each of the cDNA preparations were PCR-amplified as above using pairs of the following primers: exon 1 fwd, 5′-CGAAGCTTCCACCATGACAGCCATCATCAAAG-3′; exon 5 fwd, 5′-ATCAAACCCTTTTGTGAAGA-3′; exon 8 rev, 5′-TCTATACTGCAAATGCTATC-3′; exon 9 rev, 5′-TTGGATCCTCAGACTTTTGTAATTTG-3′. In order to confirm the quality of RNA used and to test the efficiency of the cDNA synthesis reactions, equal volumes of each of the cDNA preparations were also PCR-amplified for 16 cycles using the primers 5′-AAGGTGAAGGTCGGAGTCAA-3′ and 5′-GGACACGGAAGGCCATGCCA-3′, which amplify a 700 bp product from the cDNA of the GAPDH gene. Gel analysis confirmed that equal amounts of GAPDH product were amplified from all four cDNAs. MMAC1/PTEN RT-PCR products were analysed, purified and sequenced as above using the primers 5′-ATCAAACCCTTTTGTGAAGA-3′, 5′-ATTCTTCATACCAGGACCAG-3′, 5′-ACCAGTTCGTCCCTTTCCAG-3′ and 5′-TCTATACTGCAAATGCTATC-3′.

### Expression vectors

Full-length PTEN cDNA was generated by RT-PCR from normal lymphocyte RNA as described above using the primers 5′-CGGGATCCACCATGACAGCCATCATCAAAGA-3′ and 5′-TTGGATCCTCAGACTTTTGTAATTTG-3′, and cloned into the BamH1 site of the bicistronic expression vector pIREShyg1 (Clontech) in sense and anti-sense orientations. The G129R and G129E mutant sequences were generated from the wild-type clones by PCR mutagenesis and similarly cloned into pIREShyg1. The Flag-epitope coding sequence was added to the 5′ end of the PTEN cDNA by PCR using the primer 5′-GTGAAGCTTCCACCATGGACTACAAGGACGACGATGACAAGATGACAGCCATCATCAAAGA-3′. The gene for Enhanced Green Fluorescent Protein (EGFP) was excised from pEGFP1 (Clontech) using BamH1 and Not1 and cloned into pIREShyg1 using these enzymes.

### Prostate epithelial cell transfections

2.5 × $10^5$ cells of each line were plated in 3.5-cm wells and transfected with 1.66 μg of plasmid using the liposomal reagents Dosper (Boehringer) for PNT2, PNT1a and PC3 or Escort (Sigma) for LNCaP. For selection of stably-transfected cells, hygromycin B was added to the culture medium three days after transfection at concentrations previously optimized for each cell line to give 100% cell kill within 10 days.

### Immunocytochemistry

Cells were fixed with 4% paraformaldehyde for 15 min, permeabilized with 70% ethanol for 15 min, washed in 50 mM tris HCl pH 7.5–150 mM NaCl (TBS), and blocked in Blocking Solution (1% casein blocking agent (Boehringer)–0.1% Tween 20 in TBS). Staining was carried out with rabbit polyclonal (Upstate Biochemicals) or mouse monoclonal (Oncogene Sciences Ab-2) anti-PTEN diluted 1:200 in Blocking Solution, followed by anti-rabbit or mouse IgG-Alexa 568 conjugate (Molecular Probes) diluted 1:200 in blocking solution. FLAG-tagged PTEN was detected with an anti-FLAG epitope M2 monoclonal antibody (Sigma). Detection of F-actin was with 6 nM rhodamine-phalloidin (Sigma). The cells were then counter-stained with 1 μg $ml^{-1}$ 4′,6-diamidino-2-phenylindole (DAPI; Sigma) in TBS, washed three times for five minutes in TBS, observed for -fluorescence using a Nikon Eclipse fluorescent microscope, and fluorescent and phase contrast images captured using the Openlab program. To detect Annexin-V binding, the cultures were washed in PBS and incubated with Annexin V-Alexa568 (Boehringer, Cat. no. 1985485), used according to the manufacturer's protocol to identify apoptotic cells (Vermes et al, 1995), and the fluorescent images again captured.

### Western blotting

Cells grown in 3.5-cm wells were scraped into 250 μl buffer containing 1% SDS and 1% dithiothreitol, heated to 100°C for 10 min, analysed by SDS-polyacrylamide gel electrophoresis, and blotted to PVDF membranes (Boehringer). The membranes were incubated in Blocking Solution (as above) for 60 min, then for 3 hours in monoclonal anti-PTEN Ab-2 (Oncogene Sciences) diluted 1:1000 in Blocking Solution. After 4 × 5 min washes in

© 2000 Cancer Research Campaign 

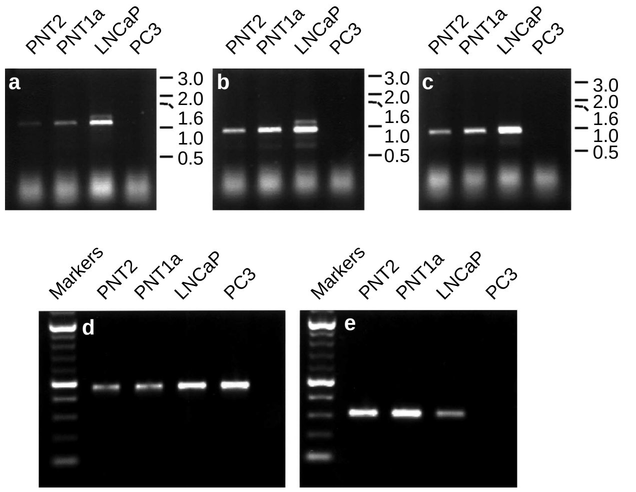

**Figure 1** Structure and expression of the MMAC1 gene in prostate carcinoma cell lines. (**a**–**c**) Expression of MMAC1 gene. RT-PCR for exons 1–9 (**a**), 1–8 (**b**) and 5–9 (**c**) of PTEN. Note the additional band from amplification of LNCaP cDNA with exons 1–9 and 1–8. The smaller band amplified by the exon1/exon 8 primer pair from PNT2, PNT1a and LNCaP cDNA results from artefactual binding of the downstream primer to an internal site in the cDNA. Marker sizes in kb are indicated to the right of each panel. The expected sizes of the RT-PCR products are: exons 1–9, 1233 bp; exons 1–8, 933 bp; exons 5–9, 920 bp. (**d**, **e**) Absence of MMAC1 exon9 in PC3 cells. Genomic PCR for exon 1 (**d**) and exon 9 (**e**) of MMAC1. The marker lanes contain a 100 bp ladder. The expected sizes of the amplified products are 484 bp for exon 1 and 331 bp for exon 9. Note the absence of the genomic DNA signal for exon 9 in the PC3 cell line consistent with a homozygous deletion.

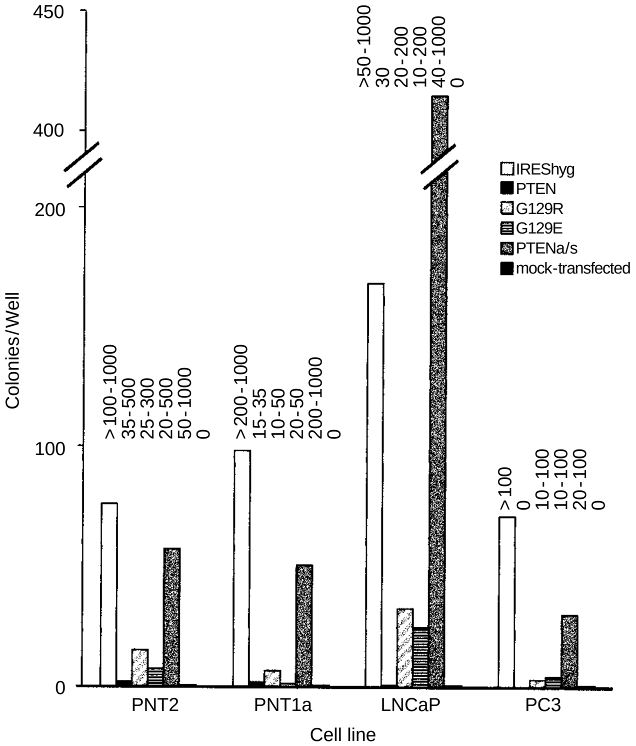

**Figure 2** Efficiency of colony formation after transfection of MMAC1 genes The vertical axis shows the number of colonies surviving after transfection with IRESHyg constructs and 14 days' selection in hygromycin in each well (average of two duplicates) after transfection of $2.5 \times 10^5$ cells with 1.66 µg of plasmid. A key to the various constructs is shown at the top right of the figure. The numbers above each bar show the range of cell numbers per hyg resistant colony. Note that no cells remained in the mock-transfected control wells. The results are representative of six independent duplicate experiments with each cell type:construct combination.

TBS, the membranes were incubated in blocking solution for 30 min, then anti-mouse-peroxidase (Sigma) diluted 1:5000 in blocking agent for 60 min. The blots were washed $4 \times 5$ min in TBS and detected by chemiluminescence (BM chemiluminescence substrate, Boehringer).

### RESULTS

### Structure and expression of the MMAC1 gene in prostate cell lines

Expression of endogenous MMAC1 mRNA in the four cell lines was measured by RT-PCR (see Figure 1). Full-length mRNA was detected in PNT1a, PNT2 and LNCaP cell lines but not in PC3. Direct sequencing of the products of the RT-PCR confirmed the expression of wt mRNA in the PNT cell lines. In LNCaP cells the RT-PCR product contained the previously reported 2bp deletion in codon 6 (Li et al, 1997), which results in an open reading frame of only 8 amino acids. In addition, we found evidence of a minor, alternatively spliced product with PCR primers spanning exons 1–8 (panel b) but not exons 5–9 (panel c). Cloning and sequencing of this product showed it to comprise the MMAC1/PTEN coding sequence (containing the 2 bp deletion in codon 6) with an insertion of 165 bases from the intron between exons 2 and 3 (corresponding to bases 80433 to 80597 inclusive of the genomic sequence of MMAC1/PTEN, NCBI gene accession no. AF067844). As the coding sequence of this cDNA still possesses the frameshift mutation in exon 6, this does not affect the essential finding that LNCaP cells do not express PTEN protein. Genomic PCR from cell line DNA confirmed the total absence of exon 9 in PC3 cell DNA, although exon 1 was present (Figure 1 d,e). Absence of detectable expression of full-length PTEN mRNA in PC3 cells thus arises from deletion of the 3′ end of the MMAC1/PTEN gene.

### PTEN expression in prostate epithelial cells using IRES expression plasmids

We employed IRES (Internal Ribosomal Entry Site) vectors to produce polycistronic mRNA in which the expression of PTEN protein was physically linked to the expression of the hygromycin (hyg) resistance gene used to derive permanent cell lines. The effectiveness of the strategy was confirmed with a hybrid vector containing EGFP-IRES-hyg: after transfection of the prostate cells and hyg selection, all of the hyg resistant cell colonies displayed green fluorescence, as shown in Figure 4a for PNT2 and PC3 cells. At 14 days after the application of selection, no colonies survived in mock-transfected cultures, while all four cell lines formed multiple colonies when transfected with IRES-hyg or PTEN antisense-IRES-hyg (see Figure 2, unfilled and grey bars). When the PTEN-IRES-hyg construct was transfected into the prostate cell lines, after 14 days of drug selection only 2–3 colonies were observed in each well containing the PNT cells, and single dispersed colonies in a few wells containing the prostate cancer cells (Figure 2, solid bars). After a further 14 days' selection, slow-growing colonies were still present with the PNT1a and PNT2 cells, whereas no survivors were seen with either PC3 or LNCaP cells.

*© 2000 Cancer Research Campaign*

### Expression of exogenous PTEN phosphatase mutants G129R and G129E

To investigate the role of the phosphatase activities of PTEN in its phenotypic effects on prostate cancer cells, the experiments were repeated with two mutant PTEN-IRES-hyg constructs: G129R, which inactivates both lipid and protein phosphatase activities, and G129E, which inactivates only the lipid phosphatase (Furnari et al, 1997, 1998; Maehama and Dixon, 1998). As shown in Figure 2 (hatched bars), transfection of both mutant MMAC1 genes resulted in a significant yield of hyg resistant colonies, which survived further selection and subculture. In terms of allowing cell survival, for PNT2 and LNCaP cells the rank order of the constructs was Empty vector > G129R > G129E >> PTEN, while PC3 cells gave slightly fewer colonies with G129R than G129E (rank order Empty vector > G129E > G129R >> PTEN). In PNT1a cells the rank order was Empty vector >> G129R > G129E ≃ PTEN (where G129E gave slightly fewer, but faster-growing, colonies than wild-type PTEN).

### Detection of PTEN immunoreactivity in transiently transfected prostate epithelial cells

Western blots of cells harvested 48 hours after transfection with the IRES constructs were probed with an anti-PTEN monoclonal antibody (Ab-2, Oncogene Sciences). As shown in Figure 3A, low levels of endogenous PTEN protein were detectable in the PNT1a and PNT2 cells, but not in the LNCaP and PC3 cells. The stronger PTEN expression observed after transfection of the three exogenous forms of PTEN was presumably due to expression of the transgenes, since cells transfected with control vector alone (pIRES-hyg) showed no equivalent stimulation. Despite efficient transfection, the PTEN levels in the tumour cell lines were significantly lower, and almost undetectable for the mutant forms in PC3 cells. PTEN expression was further investigated by immunocytochemistry, using an independent but polyclonal antibody (Upstate Biochemicals) as shown in Figure 3B. The endogenous levels of PTEN were barely visible in all but the most highly overexposed images of mock-transfected PNT2 cells (panel a). Despite achieving transfection efficiencies which resulted in 25–30% of all cells showing green fluorescence at similar times after transfection (panels q–t), wt PTEN reactivity was seen in less than 1% of transfected cultures of both normal and tumour origin (panels e to h). A slightly higher proportion of cells, but still less than 1%, showed detectable levels after transfection with mutant PTEN (G129R, panels i–1; G129E, panels m–p).

### Expression of PTEN protein in stably transfected prostatic epithelial cell clones

PNT2 and PC3 cells transfected with the EGFP-IRES-hyg construct yielded hygromycin-resistant clones which expressed uniform levels of EGFP (Figure 4A and B). In contrast, anti-PTEN immunostaining of the surviving colonies from transfection with wild type or mutant MMAC genes in MMAC-IRES-hyg constructs showed that only a small proportion of the clonal stably-transfected cells expressed detectable increases in PTEN immunoreactivity (examples shown for PNT2 with PTEN and G129R, and for PC3 cells with G129R and G129E, in Figure 4C–F). In no case did prostate tumour cells (LNCaP and PC3) transfected with wt MMAC1 survive hyg selection. Detailed examination of the cell morphologies demonstrated that stable expression of exogenous PTEN proteins at detectable levels in individual cells resulted in cell shrinkage and rounding. The severity of the morphological changes followed the same rank order as the cytotoxicity observed in the transient expressions, i.e. PTEN > G129E > G129R. The dispersed nature of the colonies is also demonstrated by the distances between the DAPI stained nuclei (Figure 4, panels C–F) compared to both the transiently transfected cultures shown in Figure 3 and the EGFP expressing PC3 clone shown in Figure 4 panel B. The change in morphology, resulting in a shrinkage of the cytoplasm round the nucleus, was also demonstrated by staining the cells with rhodamine-phallodin to target the cyto-actin cables in the cytoplasm (compare Figures 4G and 4H for the effects of PTEN in stably-transfected PNT2 cells).

**A**

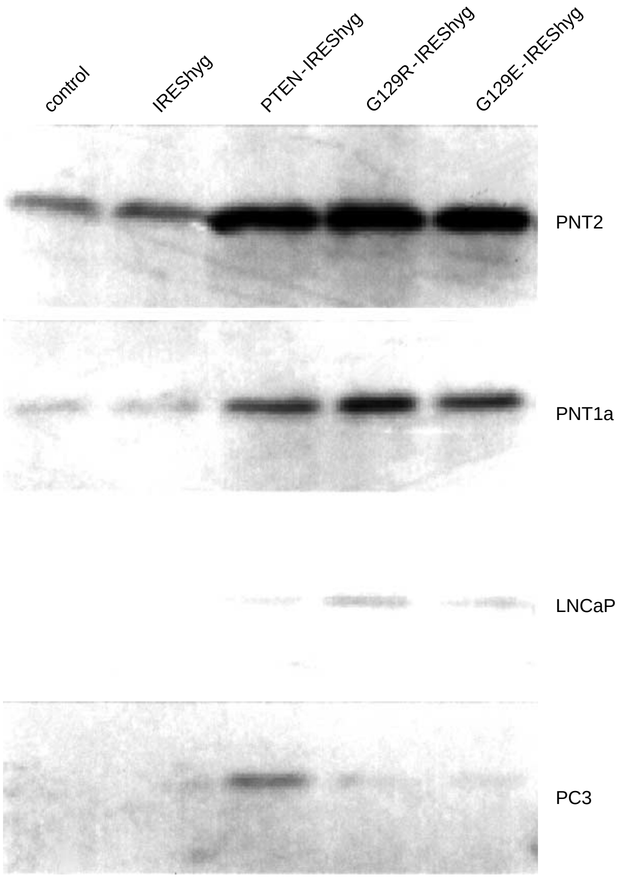

**Figure 3** Detection of PTEN protein in transiently transfected cells. (**A**) Western blotting. Western blots of protein from cells harvested 48 hours after transfection with expression constructs as indicated and probed with monoclonal anti-PTEN Ab-2 (Oncogene Sciences). PTEN immunoreactivity migrates at an approximate apparent molecular weight of 60 kD. Control cells were mock-transfected. Note the absence of PTEN protein in LNCaP and PC3 cells after mock-transfection or transfection with empty vector. (**B**) Immunocytochemistry. **(a–p):** Fluorescence immunocytochemistry of PTEN expression (red fluorescence) in transiently transfected cells: PNT2 (**a,e,i,m**), PNT1a (**b,f,j,n**), LNCaP (**c,g,k,o**) and PC3 (**d,h,l,p**). Cells were fixed and immunostained 48 hours after transfection with IRES-hyg (**a–d**), PTEN-IRES-hyg (**e–h**), G129R-IRES-hyg (**i–l**), or G129E-IRES-hyg (**m–p**). (**q–t**): EGFP Fluorescence microscopy of PNT2 (**q**), PNT1a (**r**), LNCaP (**s**) and PC3 (**t**) cells, 48 hours after transfection with EGFP-IRES-hyg. All cells are counter-stained with the DNA stain DAPI (blue fluorescence). Bar (in panel a) = 100 μm; all images are at the same magnification.

*© 2000 Cancer Research Campaign*

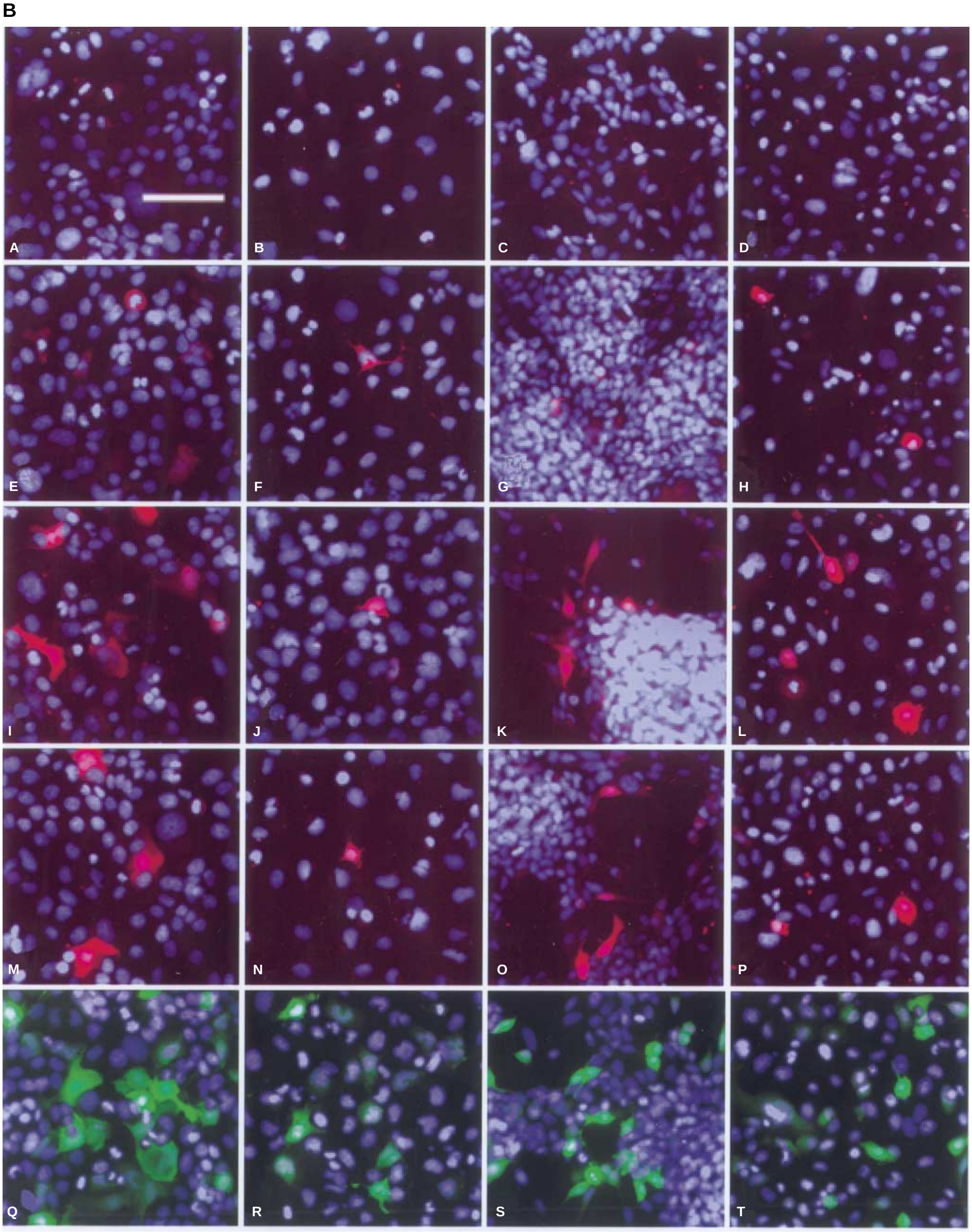

**Figure 3** continued.

*© 2000 Cancer Research Campaign*

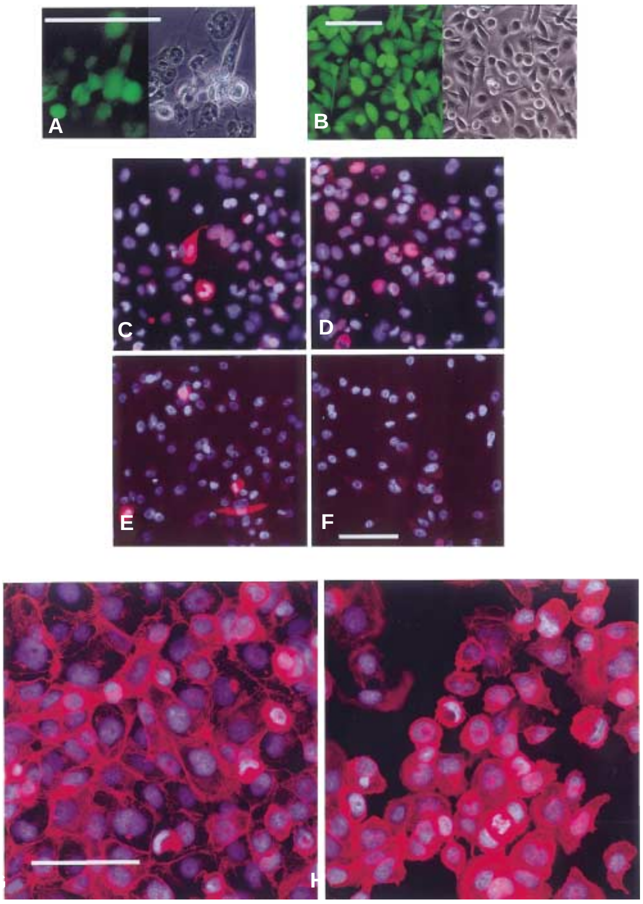

**Figure 4** Expression of PTEN protein in established transformants. (**A, B**) Uniform EGFP fluorescence in PNT2 (**A**) and PC3 (**B**) cells stably transformed with EGFP-IRES-hyg. (**C–F**): Fluorescence immunocytochemistry of PTEN expression (red fluorescence) in stably transfected cells: PNT2 cells stably transfected with PTEN-IRES-hyg (**C**) or G129R-IRES-hyg (**D**); PC3 cells stably transfected with G129R-IRES-hyg (**E**) or G129E-IRES-hyg (**F**). (**G,H**) Rhodamine-phalloidin/DAPI double staining of (**G**) prostatic epithelial cells (PNT2) stably transfected with an empty IRES-hyg vector and (**H**) with the MMAC1 gene IRES-hyg vector as shown in **C** above to be expressing exogenous PTEN protein (both lines selected with hygromycin). The altered cyto-actin patterns (red fluorescence) are a good indicator of the PTEN-induced cell rounding and loss of cell:cell and cell:substrate adhesion. All cells are counterstained with the DNA stain DAPI (blue fluorescence). White bars indicate a scale of 100 μm.

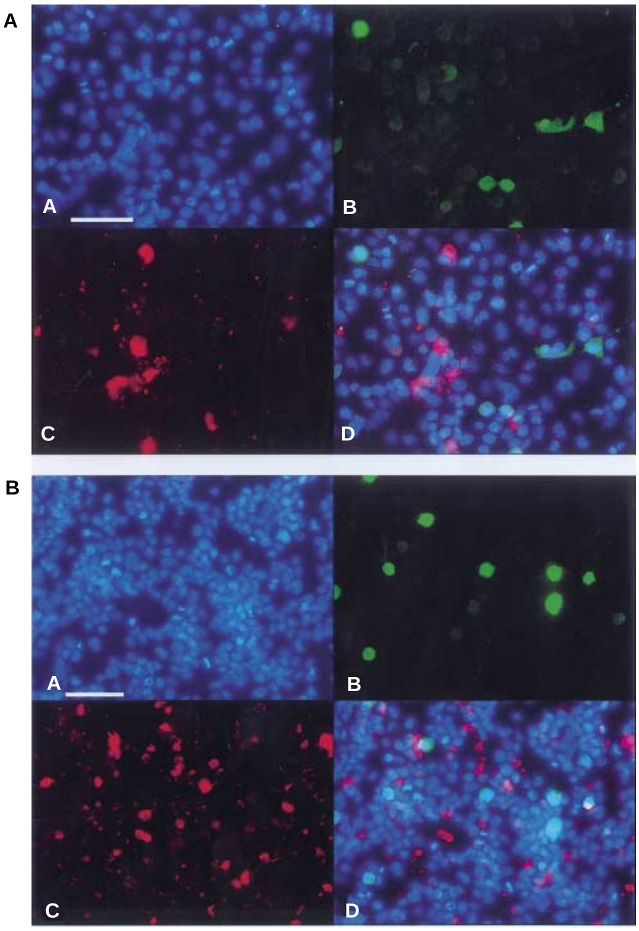

**Figure 5** Evidence of apoptosis in prostate epithelial cells expressing FLAG tagged PTEN protein. Alignment of annexin-V staining with FLAG-PTEN fluorescence in transiently transfected PNT2 (**A**) and LNCaP cells (**B**), 48 hours post transfection. The four panels show fluorescence image captures of the same field for (**A**) DAPI stain, (**B**) FLAG-tagged PTEN protein, (**C**) Annexin-V binding and (**D**) Combined image. Note that even in the cells overexpressing FLAG-PTEN, no Annexin-V binding or nuclear changes associated with apoptosis are observed. Results with untagged PTEN were identical, except that the detection of PTEN protein was limited by the sensitivity of the anti-PTEN antiserum.

### Evidence for apoptosis in prostate epithelial cells expressing PTEN and FLAG-PTEN

Biochemical evidence of the earliest signs of apoptosis in control and transfected cells was obtained from annexin-V binding assays, and counterstaining of permeabilized cell nuclei with DAPI was used to provide morphological evidence of the changes in nuclear architecture associated with apoptosis. The prostate tumour cells showed a higher than average spontaneous apoptotic rate compared to the PNT cells when assessed by Annexin-V binding (compare the red stained panels in Figures 5A (PNT2) and B (LNCaP)). However, neither transient nor stable transfection with PTEN showed a significant increase in morphological apoptosis. Fluorescence-activated cell sorting analysis of the same populations (not shown) confirmed the visual result. In addition, when the cells were incubated with annexin-V, neither co-localization of the annexin-V binding cells with PTEN expression, nor any increase in annexin-V binding cells in the stably transfected or PTEN over-expressing populations was evident. This is demonstrated in Figure 5A/B where PNT2 and LNCaP cells are shown 48 hours after transfection with a FLAG-PTEN fusion construct. Note that no nuclear morphological changes were observed in the green (anti-FLAG-PTEN stained) fluorescent cells, and no co-localization of the annexin-V (red) fluorescence with the FLAG-PTEN (green) signal can be seen in the combined images (Figures 5A and B–panel D).

### DISCUSSION

In the present study we compared prostate tumour cells, which were confirmed to be PTEN-null (Li et al, 1997), with non-tumour cell lines derived from prostatic epithelium, which we showed to express wt PTEN. In order to study the long-term effects on growth and survival of cells overexpressing MMAC1/PTEN, we used the IRES-hyg vector system (Rees et al, 1996) to ensure that all cells surviving the selection procedure expressed the transgene at least to mRNA level. Our finding that non-tumour prostatic cells can survive overexpression of MMAC1/PTEN on a long-term basis, while the tumour cells invariably fail to generate any stable transfectants, suggests that the loss of MMAC1/PTEN from tumour cells permits their survival and growth within the context of other genetic and functional changes within those cells which would be deleterious or lethal against a background of normal PTEN expression. This would be in accordance with the observation that mutation and/or deletion of MMAC1/PTEN is a late-stage phenomenon in prostate tumour progression (Dong et al, 1998), driven by selective pressures from earlier genetic aberrations, rather than an initiatory event in tumorigenesis. MMAC gene transduction has similarly been found to be selectively and rapidly cytotoxic for glioblastoma cells (Cheney et al, 1998).

Our analysis of PTEN immunocytochemistry, cytoskeletal changes, and apoptosis in MMAC-transfected cells indicated that introduction of the transgene caused alterations in cellular morphology and adhesion similar to those observed in glioblastoma cells (Davies et al, 1998; Tamura et al, 1998; Maier et al, 1999). However, we were unable to confirm the stimulation of classical apoptosis as the primary consequence of PTEN overexpression. Rather, our data support changes in cell shape followed by more rapid loss of adhesion as the primary effect (Tamura et al, 1998). Apoptosis in the detached cell populations would be a final, much later, consequence of these events. This effect has been termed anoikis in other cellular systems (Frisch and Francis, 1994; Frisch and Ruoslahti, 1997; Davies et al, 1998). The data is therefore consistent with the data reported by Davies et al (1999) in LNCaP cells, where MMAC1-induced apoptosis was detected at a much later time (after 4–6 days of PTEN expression). In our system, most of the PTEN expressing cells were lost from the tissue culture plastic surface, whether or not it had been coated with polylysine to enhance spreading and adhesion, by 4–6 days after transfection.

Western blot and immunocytochemical analysis of PTEN protein expression in cells before and after transfection with wt MMAC1/PTEN and phosphatase-defective forms demonstrate that increases in PTEN protein expression levels attained after transfection are considerably less than would be predicted on the basis of transgene mRNA level measurements. These increases are not uniform throughout the transfected cell population but are a combination of strong overexpression in a small percentage of the cells and very modest increases in PTEN levels in the majority. As normal cellular levels of full-length PTEN protein are very low, compared to relatively normal mRNA levels, it is likely that excess PTEN protein of either endogenous or exogenous origin is rapidly turned over. Thus, the individual transfected cells which express high PTEN levels may have undergone physiological changes permissive to this accumulation, such as arrest in a specific phase of the cell cycle (Furnari et al, 1998; Cheney et al, 1999; Ramaswamy et al, 1999), and loss or down-regulation of the PTEN-specific degradation machinery. Equally, these cells may represent the fraction of the transfected cells in which PTEN expression has overtaken its degradation, and are therefore at increased risk of loss from the cell layer.

Inactivation of lipid phosphatase (by the G129E mutation) or lipid and protein phosphatase (G129R mutation) activities did not completely overcome the effects of PTEN on morphology, growth or survival, as observed in glioblastoma cells by Furnari et al (1998). Other biological properties of the PTEN protein, such as interaction with cytoskeletal elements including focal adhesions, C2-binding membrane sites, or PDZ-domain proteins (Georgescu et al, 1999; Maier et al, 1999) must therefore play a role in the mechanism by which PTEN regulates cell growth and behaviour. The mechanism of these functions may be clarified by the insights into structure/function relationships afforded by the newly determined crystal structure of PTEN protein (Lee et al, 1999). The identification of functions other than phosphatase activities will also assist in the analysis of different classes of mutations in PTEN which would be predicted to result in different phenotypic effects when presenting as germline heterozygotes or as somatic mutations.

In conclusion, our results demonstrate the enhanced cytotoxicity of an exogenous MMAC1 gene in two common metastatic prostate cancer cell lines representative of androgen dependent and independent phenotypes, which did not survive long-term re-expression of the gene, particularly when compared to two prostatic epithelial cell lines of normal origin, which showed a capacity to survive MMAC1 overexpression. Were such a sensitivity to be reproducible in prostatic tissues, this differential effect of overexpression of exogenous MMAC1 between tumour and non-tumour cells might be exploited by targeting the gene to prostate cells (for example, using a prostate cell surface-specific antigen-targeted viral vector) in an expression cassette under the control of a promoter derived from a prostate-specific gene. Such an approach could offer a viable strategy for tumour therapy, even in hormone-independent advanced stage prostatic carcinomas.

### ACKNOWLEDGEMENTS

We thank Mrs Jacky Knight and Mrs Kath Ramsey for excellent technical assistance and Mrs Jo Birch for maintaining the reference database. This work was funded by Yorkshire Cancer Research.

### REFERENCES

Bergerheim USR, Kunimi K, Collins VP and Ekman P (1991) Deletion mapping of chromosome-8, chromosome-10, and chromosome-16 in human prostatic carcinoma. *Genes Chromosom Cancer* **3**: 215–220

Berthon P, Cussenot O, Hopwood L, Le Duc A and Maitland NJ (1995) Functional expression of SV40 in normal human prostatic epithelial and fibroblastic cells: differentiation pattern of non-tumorigenic cell lines. *Int J Oncol* **6**: 333–343

Boström J, Cobbers JMJL, Wolter M, Tabatabai G, Weber RG, Lichter P, Collins VP and Reifenberger G (1998) Mutation of the *PTEN (MMAC1)* tumor suppressor gene in a subset of glioblastomas but not in meningiomas with loss of chromosome arm 10q. *Cancer Res* **58**: 29–33

Cairns P, Okami K, Halachmi S, Halachmi N, Esteller M, Herman Jg Jen J, Isaacs WB, Bova GS and Sidransky D (1997) Frequent inactivation of *PTEN/MMAC1* in primary prostate cancer. *Cancer Res* **57**: 4997–5000

Cheney IW, Johnson DE, Vaillancourt MT, Avanzini J, Morimoto A, Demers GW, Wills KN, Shabram PW, Bolen JB, Tavtigian SV and Bookstein R (1998) Suppression of tumorigenicity of glioblastoma cells by adenovirus-mediated *MMAC1/PTEN* gene transfer. *Cancer Res* **58**: 2331–2334

Cheney IW, Neuteboom STC, Vaillancourt MT, Ramachandra M and Bookstein R (1999) Adenovirus-mediated gene transfer of *MMAC1/PTEN* to glioblastoma cells inhibits S phase entry by the recruitment of $p27^{kipl}$ into cyclin E/CDK2 complexes. *Cancer Res* **59**: 2318–2323

Cussenot O, Berthon P, Faille A, Berger R, Mowszowicz I, Teillac P, LeDuc A and Calvo F (1991) Immortalization of human adult normal prostatic epithelial cells by liposomes containing SV40. *J Urol* **143**: 881–886

Davies MA, Lu Y, Sano T, Fang X, Tang P, Lapushin R, Koul D, Bookstein R, Stokoe D, Yung WKA, Mills GB and Steck PA (1998) *MMAC/PTEN* in human glioma cells inhibits Akt activation and induces anoikis. *Cancer Res* **58**: 5285–5290

*© 2000 Cancer Research Campaign*

Davies MA, Koul D, Dhesi H, Berman R, Mcdonnell TJ, Mcconkey D, Yung WKA and Steck PA (1999) Regulation of Akt/PKB activity, cellular growth, and apoptosis in prostate carcinoma cells by *MMAC/PTEN*. *Cancer Res* **59**: 2551–2556

Dong JT, Sipe TW, Hyytinen ER, Li, CL, Heise C, McClintock DE, Grant CD, Chung LWK and Frierson HF Jr (1998) *PTEN/MMAC1* is infrequently mutated in pT2 and pT3 carcinomas of the prostate. *Oncogene* **17**: 1979–1982

Frisch SM and Francis H (1994) Disruption of epithelial cell-matrix interactions induces apoptosis *J Cell Biol* **124**: 619–626

Frisch SM and Ruoslahti E (1997) Integrins and anoikis. *Curr Opin Cell Biol* **9**: 701–706

Furnari FB, Lin H, Huang HJS and Cavenee WK (1997) Growth suppression of glioma cells by PTEN requires a functional phosphatase catalytic domain. *Proc Natl Acad Sci USA* **94**: 12479–12484

Furnari FB, Huang H-JS and Cavanee WK (1988) The phosphoinositol phosphatase activity of PTEN mediates a serum-sensitive $G_1$ growth arrest in glioma cells. *Cancer Res* **58**: 5002–5008

Georgescu M-M, Kirsch KH, Akagi T, Shishido T and Hanafusa H (1999) The tumor-suppressor activity of PTEN is regulated by its carboxyl-terminal region. *Proc Natl Acad Sci USA* **96**: 10182–10187

Gray IC, Phillips SMA, Lee SJ, Neoptolemos JP, Weissenbach J and Spurr NK (1995) Loss of the chromosomal region 10q23–25 in prostate cancer. *Cancer Res* **55**: 4800–4803

Gray IC, Stewart LMD, Phillips SMA, Hamilton JA, Gray NE, Watson GJ, Spurr NK and Snary D (1998) Mutation and expression analysis of the putative prostate tumour-suppressor gene PTEN. *Br J Cancer* **78**: 1296–1300

Guldberg P, Straten PT, Birck A, Ahrenkiel V, Kirkin AF and Zeuthen J (1997) Disruption of the *MMAC1/PTEN* gene by deletion or mutation is a frequent event in malignant melanoma. *Cancer Res* **57**: 3660–3663

Ittmann M (1996) Allelic loss on chromosome 10 in prostate adenocarcinoma. *Cancer Res* **56**: 2143–2147

Latil A and Lidereau R (1998) Genetic aspects of prostate cancer. *Virchows Archiv an International Journal of Pathology* **432**: 389–406

Lee J-O, Yang H, Georgescu M-M, Di Cristofano A, Maehama T, Shi Y, Dixon JE, Pandolfi P and Pavletich NP (1999) Crystal structure of the PTEN tumor suppressor: implications for its phosphoinositide phosphatase activity and membrane association. *Cell* **99**: 323–334

Li J, Yen C, Liaw D, Podsypanina K, Bose S, Wang SI, Puc J, Miliaresis C, Rodgers L, Mccombie R, Bigner SH, Giovanella BC, Ittmann M, Tycko B, Hibshoosh H, Wigler MH and Parsons R (1997) *PTEN*, a putative protein tyrosine phosphatase gene mutated in human brain, breast, and prostate cancer. *Science* **275**: 1943–1947

Macintosh CA, Stower M, Reid N and Maitland NJ (1998) Precise microdissection of human prostate cancers reveals genotypic heterogeneity. *Cancer Res* **58**: 23–28

Maehama T and Dixon JE (1998) The tumor suppressor, PTEN/MMAC1, dephosphorylates the lipid seond messenger, phosphatidylinositol 3,4,5-trisphosphate. *J Biol Chem* **273**: 13375–13378

Maier D, Jones G, Li XW, Schönthal AH, Gratzl O, Van Meir EG and Merlo A (1999) The PTEN lipid phosphatase domain is not required to inhibit invasion of glioma cells. *Cancer Res* **59**: 5479–5482

Myers MP, Stolarov JP, Eng C, Li J, Wang SI, Wigler MH, Parsons R and Tonks NK (1997) P-TEN, the tumor supressor from human chromosome 10q23, is a dual-specificity phosphatase. *Proc Natl Acad Sci USA* **94**: 9052–9057

Myers MP, Pass I, Batty IH, Van Der Kaay J, Stolarov JP, Hemmings BA, Wigler MH, Downes CP and Tonks NK (1998) The lipid phosphatase activity of PTEN is critical for its tumor supressor function. *Proc Natl Acad Sci USA* **95**: 13513–13518

Pesche S, Latil A, Muzeau F, Cussenot O, Fournier G, Longy M, Eng C and Lidereau R (1998) *PTEN/MMAC1/TEP1* involvement in primary prostate cancers. *Oncogene* **16**: 2879–2883

Ramaswamy S, Nakamura N, Vazquez F, Batt DB, Perera S, Roberts TM and Sellers WR (1999) Regulation of G1 progression by the *PTEN* tumor suppressor protein is linked to inhibition of the phosphatidylinositol 3-kinase/Akt pathway. *Proc Natl Acad Sci USA* **96**: 2110–2115

Rees S, Coote J, Stables J, Goodson S, Harris S and Lee MG (1996) Bicistronic vector for the creation of stable mammalian cell lines that predisposes all antibiotic-resistant cells to express recombinant protein. *BioTechniques* **20**: 102–110

Rhei E, Kang L, Bogomolniy F, Federici MG, Borgen PI and Boyd J (1997) Mutational analysis of the putative tumor suppressor gene *PTEN/MMAC1* in primary breast carcinomas. *Cancer Res* **57**: 3657–3659

Steck PA, Pershouse MA, Jasser SA, Yung WKA, Lin H, Ligon AH, Langford LA, Baumgard ML, Hattier T, Davis T, Frye C, Hu R, Swedlund B, Teng DHF and Tavtigian SV (1997) Identification of a candidate tumour suppressor gene, *MMAC1*, at chromosome 10q23.3 that is mutated in multiple advanced cancers. *Nature Genetics* **15**: 356–362

Suzuki H, Freije D, Nusskern DR, Okami K, Cairns P, Sidransky D, Isaacs WB and Bova GS (1998) Interfocal heterogeneity of *PTEN/MMAC1* gene alterations in multiple metastatic prostate cancer tissues. *Cancer Res* **58**: 204–209

Tamura M, Gu J, Matsumoto K, Aota S, Parsons R and Yamada KM (1998) Inhibition of cell migration, spreading and focal adhesions by tumor suppressor PTEN. *Science* **280**: 1614–1617

Tashiro H, Blazes MS, Wu R, Cho KR, Bose S, Wang SI, Li, J, Parsons R and Ellenson LH (1997) Mutations in PTEN are frequent in endometrial carcinoma but rare in other common gynecological malignancies. *Cancer Res* **57**: 3935–3940

Trybus TM, Burgess AC, Wojno KJ, Glover TW and Macoska JA (1996) Distinct areas of allelic loss on chromosomal regions 10p and 10q in human prostate cancer. *Cancer Res* **56**: 2263–2267

Vermes I, Haanen C, Steffensnakken H and Reutelingsperger C (1995) A novel assay for apoptosis – flow cytomeric detection of phosphatidylserine expression on early apoptotic cells using fluorescein-labeled annexin-V. *J Immunol Methods* **184**: 39–51

© 2000 Cancer Research Campaign
